# Angiomyolipoma of the broad ligament

**DOI:** 10.4322/acr.2020.173

**Published:** 2020-07-01

**Authors:** Satya Dutta, Evarisalin Marbaniang, Biswajit Dey, Bifica Sofia Lyngdoh, Vandana Raphael

**Affiliations:** a North Eastern Indira Gandhi Regional Institute of Health and Medical Sciences (NEIGRIHMS), Department of Pathology. Shillong, India.

**Keywords:** Angiomyolipoma, Immunohistochemistry, Adipose Tissue

## Abstract

Angiomyolipoma is a benign mesenchymal neoplasm of the renal parenchyma, accounting for 1% of all renal parenchymal tumors. However, this entity may rarely occur in extrarenal sites. Extrarenal angiomyolipoma has been documented in various sites of the body, but angiomyolipoma of the broad ligament was reported in only two cases. We report the reputed third case of angiomyolipoma of the broad ligament in a 33-year-old female, who presented clinically with abdominal distension. With a working diagnosis of low-grade neoplasm, an *en-bloc* excision of the left broad ligament mass was performed. Based on histopathology and immunohistochemistry, a diagnosis of the classical variant of angiomyolipoma of the left broad ligament was made. The post-operative period was uneventful with no recurrence after 6 months of follow-up.

## CASE REPORT

**Figure 1:**
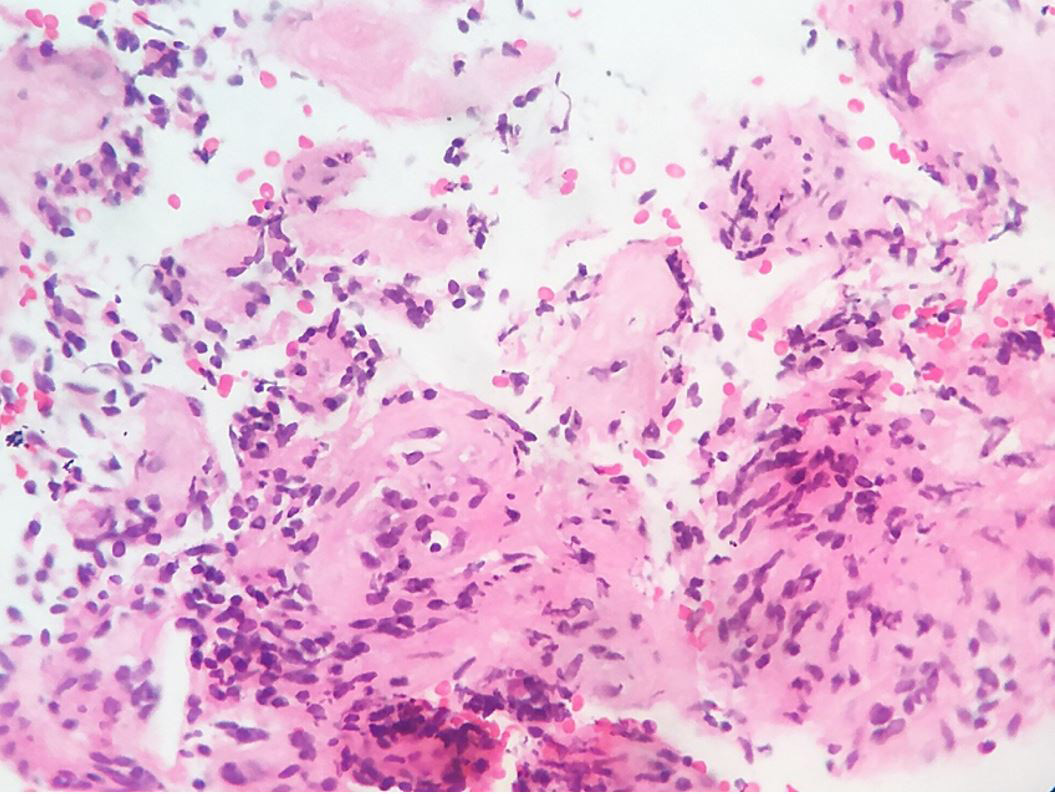
Photomicrograph of the Tru-cut biopsy showing mostly spindle cells along with many proliferating vascular channels (H & E, 40x).

A 33-year-old female patient sought medical care complaining of abdominal distension accompanied by feet edema. She referred abdominal pain and decreased urine output over the last 4 months. She denied any other symptom when actively questioned. Her past medical history was unremarkable and also denied smoking or alcohol consumption. On physical examination, the abdomen was distended with mild tenderness along with a large mass palpable in the hypogastric region. The remaining physical examination was normal. The laboratory workup and chest X-ray were normal. However, the pelvic ultrasonography (USG) depicted a large heteroechoic lesion in the left broad ligament extending to the pouch of Douglas, measuring 12×13.2 cm with internal vascularity. There was no lymphadenopathy; however, minimal ascites was noted. On contrast-enhanced computed tomography (CECT), a well-defined abdominopelvic soft tissue density lesion centered in the left broad ligament, with subtle heterogeneous enhancement on the post-contrast study, measuring 21.2×20×10 cm displacing the bowel loops superiorly and laterally, was noted. On per speculum examinations, the cervix was pulled up, and the vagina was healthy. On per vaginal examination, the uterus was bulky, and the pouch of Douglas was full. Cytological examination of the ascitic fluid examination was negative for malignancy. The USG-guided fine needle aspiration cytology attempted from the mass showed occasional stromal fragments, proliferating vascular fragments, and inflammatory cells in a hemorrhagic background. The USG-guided Tru-cut biopsy from the mass showed scant tissue bits with spindle cells along with many proliferating vascular channels suggestive of low-grade spindle cell neoplasm (Figure 1).

**Figure 2:**
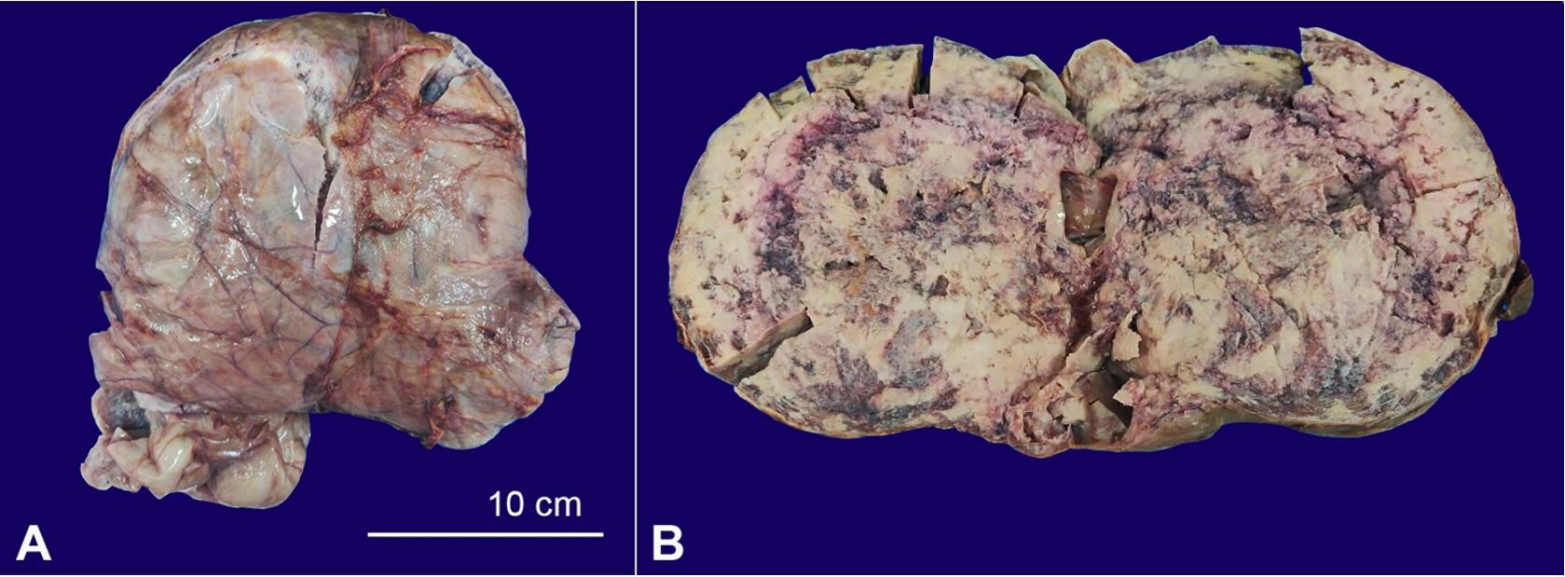
**A** – Gross view of the capsulated and lobulated mass; **B** – cut surface was solid grey-white with areas of hemorrhage.

Due to the working diagnosis of a low-grade neoplasm, an *en-bloc* excision of the left broad ligament mass was performed. Per-operatively the mass was in the left broad ligament extending to the pouch of Douglas. The mass did not involve the uterus, the bilateral ovaries, and fallopian tubes. Grossly the mass was lobulated, encapsulated, and measured 21×20×10 cm (Figure 2A). The cut surface was solid grey-white with areas of hemorrhage and few cystic areas filled with a mucoid material (Figure 2B).

**Figure 3:**
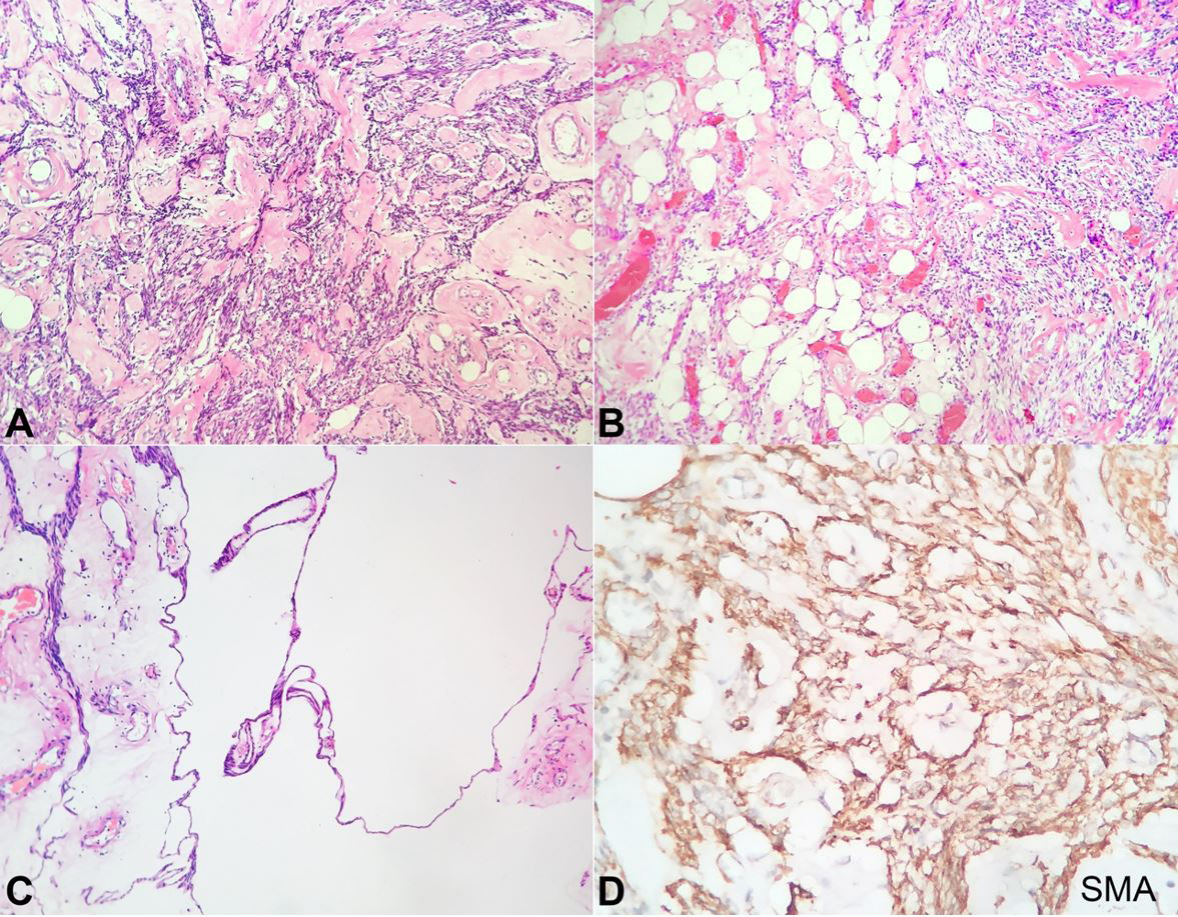
Photomicrographs of the surgical specimen. **A –** showing thickened hyalinized median caliber blood vessels with many intersecting bundles of smooth muscle (H&E, 20X); **B** – Interspersed mature clusters of mature adipose tissue (H&E, 20X); **C** – Areas of myxoid degeneration with cystic spaces not lined by epithelium (H&E, 10X); **D** – SMA diffusely positive in smooth muscle component (20x)

The histopathological examination showed many thickened hyalinized median caliber blood vessels with many intersecting bundles of smooth muscle, which at some places seemed to radiate out from the walls of the blood vessels (Figure 3A). Interspersed mature clusters of mature adipose tissue were seen (Figure 3B). There were areas of myxoid degeneration with cystic spaces, which were not lined by epithelium (Figure 3C). Mitosis was rare. Immunohistochemically, smooth muscle actin (SMA) was diffusely positive in smooth muscle component and vessel wall, CD 34 was diffusely positive in the endothelial cells highlighting the vessels, S100 was positive in the mature adipose tissue component (Figure 3D). However, HMB-45 was negative. Correlating with histology and immunohistochemistry, the diagnosis of the classical variant of angiomyolipoma (AML) of the left broad ligament was made. The post-operative period was uneventful, and the patient was discharged on the 7^th^ post-operative day. The patient had no recurrence after 6 months of follow-up.

## DISCUSSION

Angiomyolipoma (AML) is an uncommon benign mesenchymal neoplasm of unknown origin, comprising of mature adipose tissue, muscle fibers, and thickened-wall blood vessels.^1^ ^,^ ^2^ In contrast to renal AML, extrarenal AML are rarer and have been documented in the liver, lung, retroperitoneum and female genital tract.^3^ ^-^ ^6^ AML is a relatively rare benign tumor appearing in about 0.3% of the general population and accounts for 3% of all the solid renal masses.^7^ AML is part of the PEComa group of tumors.^8^

The PEComa group of tumors includes mainly the AML, lymphangioma, pulmonary clear cell “sugar” tumor (CCST), lymphangioleiomyomatosis (LAM), and the clear cell myomelanocytic tumor of the falciform ligament/ligamentum teres of various anatomical sites.^8^ In a meta-analysis of PEComas of the female genital tract, Liu et al.^8^ showed only 5 cases located in the broad ligament. In addition, PEComas of the broad ligament have also been reported by other authors.^9^ ^-^ ^13^

Few cases of AML involving the female genital tract have been reported, but AML of broad ligaments is very rare.^14^ ^,^ ^15^ A detailed search in Google Scholar and PubMed using the keywords ‘Angiomyolipoma’ and ‘Broad ligament’ yielded only two cases.^14^ ^,^ ^15^ AML may or may not be associated with tuberous sclerosis.^16^ In the present case, the patient did not have any signs or symptoms of the tuberous sclerosis complex.^17^ Usually, extrarenal AML is not associated with tuberous sclerosis.^16^

Chopra et al.^14^ documented the presence of the interspersed epithelium lined cystic areas consistent with the paramesonephric (Mullerian) cystic remnants. The case reported by Shakuntala et al.^15^ did not find entrapment of the paramesonephric remnants. In the present case, the mass was grossly solid, with areas of cyst formations that were filled with mucoid material. Microscopically, these cysts were not lined by any epithelium, therefore, ruling out their entrapment of paramesonephric duct remnants. Histopathologically, AML has all three components, namely myoid component, vascular component, and adipose tissue component in variable proportion.^1^ This unique histopathological appearance of AML helps in ruling out the other differential diagnoses like lipomatosis, lipoma, lipoblastoma, leiomyoma, lipoleiomyoma, and liposarcoma, which have only one or two components.^15^ ^,^ ^18^

Immunohistochemically the myoid component was positive for smooth muscle actin antibody and negative for HMB-45. The vascular component was positive for CD 34, and the adipose tissue component was positive for S-100. Shakuntala et al.^15^ and Yaegashi et al.^19^ reported cases of extrarenal AML of the female genital tract with a negative reaction to HMB-45.

The classical variant of AML is a benign lesion with locally aggressive behavior, recurrence potential; however, metastasis does not occur.^3^ The epithelioid AML is a variant with malignant potential.^3^ The present case was a classical variant of AML. Therefore, complete surgical excision is the treatment of choice.

AML arising from the broad ligament is very rare. As far as we could get, we only found two cases described in the English literature. Although rare, AML should be kept in the differential diagnoses of the broad ligament masses, including those of malignant behavior. Since AML have recurrence potential, they should also be differentiated from the benign differentials. The characteristic histopathology comprising of mature adipose tissue, muscle fibers, and thickened-wall blood vessels with a panel of immunohistochemical markers is required for their accurate diagnosis. Considering that the AML is a benign entity, but with recurrence potential, complete surgical excision is desirable.

## References

[1] Tsutsumi M, Yamauchi A, Tsukamoto S, Ishikawa S (2001). A case of angiomyolipoma presenting as a huge retroperitoneal mass. Int J Urol.

[2] Minja EJ, Pellerin M, Saviano N, Chamberlain RS (2012). Retroperitoneal extrarenal angiomyolipomas: an evidence-based approach to a rare clinical entity. Case Rep Nephrol.

[3] Eble JN (1998). Angiomyolipoma of kidney. Semin Diagn Pathol.

[4] Goodman ZD, Ishak KG (1984). Angiomyolipomas of the liver. Am J Surg Pathol.

[5] Katz DA, Thom D, Bogard P, Dermer MS (1984). Angiomyolipoma of the fallopian tube. Am J Obstet Gynecol.

[6] Laffargue F, Giacalone PL, Charpin C, Lachard A (1993). Uterine angiomyolipoma associated with pregnancy. Gynecol Oncol.

[7] Nelson CP, Sanda MG (2002). Contemporary diagnosis and management of renal angiomyolipoma. J Urol.

[8] Liu CH, Chao WT, Lin SC, Lau HY, Wu HH, Wang PH (2019). Malignant perivascular epithelioid cell tumor in the female genital tract: preferred reporting items for systematic reviews and meta-analyses. Medicine.

[9] Mathew M, Nayal B, Rao L, Nagel B (2016). Broad ligament perivascular epithelioid cell tumor (PEComa) of uncertain malignant potential. Turk Patoloji Derg.

[10] Ryś J, Karolewski K, Pudełek J (2008). perivascular epithelioid cell tumor (PEComa) of the falciform/broad ligament. Pol J Pathol.

[11] Kim HJ, Lim SJ, Choi H, Park K (2006). Malignant clear-cell myomelanocytic tumor of broad ligament: a case report. Virchows Arch.

[12] Fink D, Marsden DE, Edwards L, Camaris C, Hacker NF (2004). Malignant perivascular epithelioid cell tumor (PEComa) arising in the broad ligament. Int J Gynecol Cancer.

[13] Bonis RT, Domínguez TP, Calatayud FR, Jiménez EC, Piris MA, López-Lucendo NJ (2008). Pelvic PEComa of the broad ligament with lymph node metastases: case report and review of the literature. Arch Esp Urol.

[14] Chopra R, Al-Mulhim AR, Hashish H (2003). Parametrial angiomyolipoma with multicystic change. Gynecol Oncol.

[15] Shakuntala PN, Shilpashree M, Geethanjali S, Sharma SK (2012). Acute abdomen as an unusual presentation of broad ligament angiomyolipoma A case report and review of literature. Indian J Surg Oncol.

[16] Tseng CA, Pan YS, Su YC, Wu DC, Jan CM, Wang WM (2004). Extrarenal retroperitoneal angiomyolipoma: case report and review of the literature. Abdom Imaging.

[17] Curatolo P, Maria BL (2013). Tuberous sclerosis. Handb Clin Neurol.

[18] Dutta S, Dey B, Chanu SM, Raphael V, Khonglah Y (2019). Uterine lipoleiomyoma in peri- and post-menopausal women: a report of three cases. Clin Cancer Investig J.

[19] Yaegashi H, Moriya T, Soeda S, Yonemoto Y, Nagura H, Sasano H (2001). Uterine angiomyolipoma: case report and review of the literature. Pathol Int.

